# Identification of Key Factors in Cartilage Tissue During the Progression of Osteoarthritis Using a Non-targeted Metabolomics Strategy

**DOI:** 10.1007/s43657-023-00123-z

**Published:** 2024-03-10

**Authors:** Shiyu Sun, Minghui Chen, Tingting Zhang, Yanyan Wang, Weijun Shen, Tao Zhang, Jian Liu, Haidan Lan, Jianyuan Zhao, Fuqing Lin, Xuan Zhao

**Affiliations:** 1 Department of Anesthesia, Shanghai Tenth People’s Hospital, Tongji University School of Medicine, 301 Middle Yanchang Road, Shanghai, 200072 China; 2 Institute for Developmental and Regenerative Cardiovascular Medicine, MOE-Shanghai Key Laboratory of Children’s Environmental Health, Xinhua Hospital, Shanghai Jiao Tong University School of Medicine, 1665 Kongjiang Road, Shanghai, 200092 China

**Keywords:** Non-targeted metabolomics, Osteoarthritis, Progression, Kellgren–Lawrence grade

## Abstract

This research was to reveal the key factors in the progression of osteoarthritis (OA) using non-targeted metabolomics and to find targeted therapies for patients with OA. Twenty-two patients with knee OA scheduled for total knee arthroplasty were divided into two groups: Kellgren–Lawrence (KL) grade 3 (*n* = 16) and grade 4 (*n* = 6), according to plain X-rays of the knee. After the operation, the cartilages of femur samples were analyzed using non-targeted metabolomics. When compared with grade 3 patients, the levels of choline, 2-propylpiperidine, rhamnose, and monomethyl glutaric acid were higher; while 1-methylhistamine, sphingomyelin (SM) (d18:1/14:0), zeranol, 3- (4-hydroxyphenyl)-1-propanol, 5-aminopentanamide, dihydrouracil, 2-hydroxypyridine, and 3-amino-2-piperidone were lower in grade 4 patients. Furthermore, some metabolic pathways were found to be significantly different in two groups such as the pantothenate and coenzyme A (CoA) biosynthesis pathway, the glycerophospholipid metabolism pathway, histidine metabolism pathway, lysine degradation pathway, glycine, serine and threonine metabolism pathway, fructose and mannose metabolism pathway, the pyrimidine metabolism pathway, and beta-alanine metabolism pathway. This work used non-targeted metabolomics and screened out differential metabolites and metabolic pathways, providing a reliable theoretical basis for further study of specific markers and their specific pathways in the progression of OA.

**Supplementary Information:**

The online version contains supplementary material available at 10.1007/s43657-023-00123-z.

## Introduction

Osteoarthritis (OA) is common in elderly patients and leads to impaired joint function, but the pathogenesis of OA is still poorly understood. The knee is the joint most affected by OA, and the incidence of knee OA in people over the age of 65 is about 12% in the United States (Lai et al. 2019; Wallace et al. 2017; Lee et al. 2019; Yamada et al. 2019). OA causes edema and malformation of the joint and affects patients' quality of life (Belluzzi et al. 2019; Harikesavan et al. 2019). The biomechanical function of articular cartilage is to provide structural support and resistance to deformation (Wachowski et al. 2012; Tsukuda et al. 2015). Since the inner structure of the joint and its interaction with cellular factors are very complicated, the pathology of OA is still not clear.

Metabolomics which is based on the application of nuclear magnetic resonance and mass spectrometry (MS) is a field of life science which has rapidly evolved in recent years (Nicholson et al. 1999; Tian et al. 2016). Nuclear magnetic resonance spectroscopy is a valuable technique since it is non-invasive, non-destructive, and highly reproducible, and has quantitative capabilities (Crook and Powers 2020). However, it has limited sensitivity and dynamic range. MS has the advantages of a superior sensitivity, good selectivity, and strong specificity (Tian et al. 2016; Siddiqui et al. 2020). Qualitative and quantitative analysis of small molecular metabolites (< 1500 Da) can be carried out using metabolomics which can also interpret gene function and reveal various endogenous physiological and biochemical reactions. Metabolomics is now widely used in a variety of areas such as disease diagnosis, life sciences, toxicology, drug research and development (Song et al. 2020; Tian et al. 2020; Li et al. 2020; Wang et al. 2021; Zhang et al. 2022; Zhao et al. 2023). It has been applied to investigate special markers and pathways of disease (Carlson et al. 2018). In our study, we used metabolomics to reveal markers and pathways in the progression of OA.

## Materials and Methods

### Patient Enrollment

This study was approved by the ethics committee of Shanghai Tenth People's Hospital of Tongji University (Shanghai, China, SHSY-IEC-4.1/21-241/01) and carried out in 2021. All patients gave their written informed consent before the trial. It was registered at ClinicalTrials.gov (ChiCTR2100051396, 2021.09.22). Inclusion criteria were patients aged 60–75 years with OA, scheduled for a unilateral total knee arthroplasty (TKA). Exclusion criteria were rheumatoid arthritis, osteonecrosis of the femoral head, periarticular fracture, immunotherapy or analgesic therapy within four weeks, infection, neuroarthropathy, acromegaly, osteochondroma, knee arthroscopy performed within the previous one year, metabolic diseases such as diabetes, intra-articular injection or systemic (oral, intravenous or intramuscular injection) steroid drugs in the previous six months.

## Methods

### Trial Design

Twenty-two enrolled patients underwent an X-ray scan of the affected knee before surgery. The patients were grouped according to the Kellgren–Lawrence (KL) classification system. Grade 1: doubtful narrowing of the joint space with possible osteophyte formation; grade 2: possible narrowing of the joint space with definite osteophyte formation; grade 3: definite narrowing of the joint space, moderate osteophyte formation, some sclerosis and possible deformity of bony ends; grade 4: large osteophyte formation, severe narrowing of the joint space with marked sclerosis, and definite deformity of the bone ends. Cartilages of femur samples from these 22 patients were collected after TKA operation, wrapped with wet sterile gauze, and preserved in a − 80 °C freezer.

### Metabolite Extraction Method

Bone samples were thawed on ice and were homogenized effectively using a grinding machine. The following steps were performed by Shanghai Biotree Biotech Co., Ltd (Shanghai, China). Fifty milligrams of sample were weighed into an Eppendorf tube after liquid nitrogen grinding, and 1000 μL extract solution was added. Then, the samples were homogenized at 35 Hz for four min and sonicated for five min in an ice-water bath. The homogenization and sonication cycle was repeated three times. Then the samples were incubated for one hour at − 40 ℃ and centrifuged at 12,000 rpm for 15 min at 4 ℃. The resulting supernatant was transferred to a fresh glass vial for analysis. The quality control (QC) sample was prepared by mixing equal volumes of supernatants from all of the samples. The samples were randomized in their injection order and four QC samples were injected. The data were normalized with the internal standard.

### Chromatographic Parameters

Liquid chromatography/tandem MS(LC–MS/MS) analyses were performed using an ultra-high-performance liquid chromatography (UHPLC) system (Vanquish, Thermo Fisher Scientific, Waltham, MA, USA) with an ultra-performance liquid chromatography (UPLC) BEH amide column (2.1 × 100 mm, 1.7 μm) coupled to a Q Exactive HFX mass spectrometer (Orbitrap MS, Thermo Fisher Scientific).

### MS Parameters

A Q Exactive HFX mass spectrometer was used for its ability to acquire MS/MS spectra in information-dependent acquisition mode in the control of the acquisition software (Xcalibur, Thermo Fisher Scientific). In this mode, the acquisition software continuously evaluates the full scan MS spectrum.

### Data Processing

The raw data were converted to the mzXML format using ProteoWizard (Palo Alto, CA, USA) and processed using an in-house program, which was developed using R and based on XCMS, for peak detection, extraction, alignment, and integration (Darren et al. 2008; Colin et al. 2006).

### Statistical Analysis

The data were analyzed using univariate statistical analysis, multivariate statistical analysis, and orthogonal partial least squares-discriminant analysis (OPLS-DA), as appropriate. Univariate statistical analysis was performed using Student's *t* test and multivariate statistical analysis was performed using principal component analysis (PCA). *p* < 0.05 represents statistical significance for all analyses. The screening criteria of differential metabolites is that the variable importance in projection (VIP) of the first principal component in the OPLS-DA model is greater than 1 or *p* < 0.05 in Student's* t* test. Kyoto encyclopedia of genes and genomes (KEGG) annotation analysis found the pathways involved in all differential metabolites.

## Results

### Characteristics of Patients in the Two Groups

**Fig. 1:**
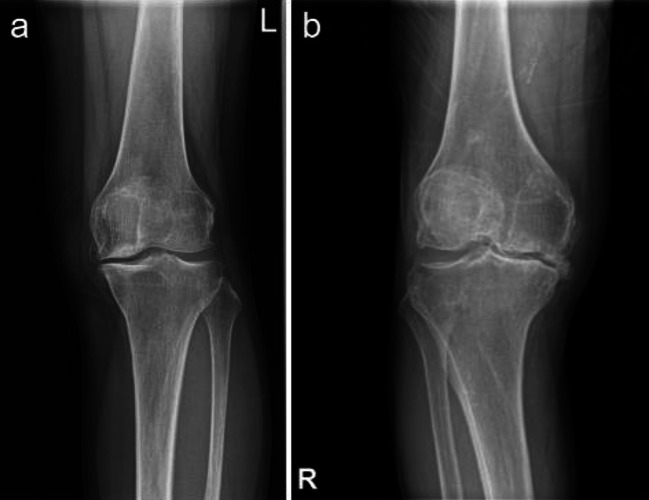
Plain X-ray of the knee, left one represents KL grade 3 a and right one represents KL grade 4 b

Twenty-two patients were grouped into two groups according to the KL classification system (Kellgren et al. 1957): 16 patients were classified as grade 3 and six as grade 4 (Fig. 1). There were no statistically significant differences in age, gender, body mass index (BMI), hypertension prevalence, or coronary heart disease prevalence between the two groups. There were no comorbidities such as gout or osteoporosis in either group (Table 1).

**Table 1:** Basic situation of patients in two groups

Variable	Patients in grade 3 (*n* = 16)	Patients in grade 4 (*n* = 6)	*p*
Age (years)	67.81 ± 3.71	68.33 ± 3.72	0.77
Female/male	11/5	3/3	0.42
BMI (kg/m^2^)	27.51 ± 2.56	27.23 ± 1.77	0.81
Hypertension	9 (56%)	4 (67%)	0.65
Coronary heart disease	8 (50%)	2 (33%)	0.48
Gout	0	0	NA
Osteoporosis	0	0	NA

### Comparison of Metabolites between the Two Groups

**Fig. 2:**
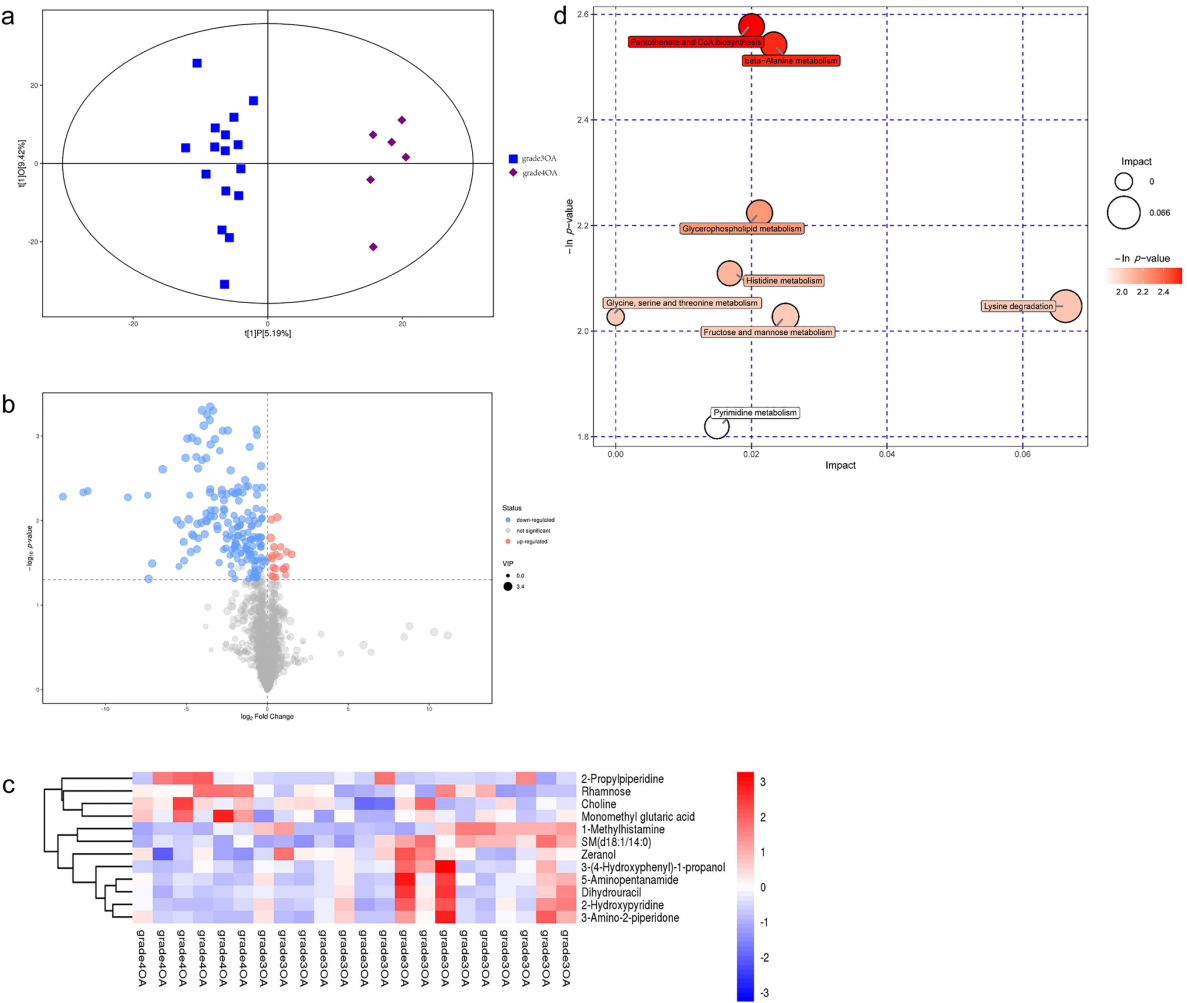
**a** Score scatter plot of OPLS-DA model for grade 4 group and grade 3 group. **b** Volcano plot for grade 4 group and grade 3 group. **c** Compared to grade 3 group patients, 2-propylpiperidine, rhamnose, choline, and monomethyl glutaric acid were significantly up-regulated; while 1-methylhistamine, SM (d18:1/14:0), zeranol, 3- (4-hydroxyphenyl)-1-propanol, 5-aminopentanamide, dihydrouracil, 2-hydroxypyridine and 3-amino-2-piperidone were significantly down-regulated in grade 4 group patients. **d** Pantothenate and CoA biosynthesis pathway, beta-alanine metabolism pathway, glycerophospholipid metabolism pathway, histidine metabolism pathway, lysine degradation pathway, glycine, serine and threonine metabolism pathway, fructose and mannose metabolism pathway, and pyrimidine metabolism pathway were statistically significant between two groups

The OPLS-DA model exhibited a clear and distinctive clustering between the two groups. It could be seen from the results of the OPLS-DA score map that the two groups of samples were very significantly distinguished, and the samples were all within the 95% confidence zone (Inside the Hotelling's T-squared ellipse) (Fig. 2a). Each point in the volcano plot represented a metabolite, and the size of the scatter point represented the VIP value of the OPLS-DA model. The larger the scatter point, the greater the VIP value. Scattered colors represented the final screening results. Significantly up-regulated metabolites were shown in red, significantly down-regulated metabolites were shown in blue and non-significantly different metabolites were shown in gray (Fig. 2b). A variety of features were detected in each sample of the two groups in positive ion mode and negative ion mode (Table 2a). Levels of 12 metabolites were significantly different between the two groups (Fig. 2c). When compared to grade 3 group patients, 2-propylpiperidine, rhamnose, choline, and monomethyl glutaric acid were significantly up-regulated while 1-methylhistamine, sphingomyelin (SM) (d18:1/14:0), zeranol, 3-(4-hydroxyphenyl)-1-propanol, 5-aminopentanamide, dihydrouracil, 2-hydroxypyridine and 3-amino-2-piperidone were significantly down-regulated in grade 4 group patients.

**Table 2:** **a** The information of positive metabolites in two groups

Metabolite	RT (s)	*m/z*	Mean grade 4	Mean grade 3	*p*
2-Propylpiperidine	224.54	128.1436	0.175750055	0.142517243	0.028^a^
Rhamnose	214.81	163.0604	0.160204593	0.071438394	0.006^a^
Choline	273.29	104.1072	0.075694853	0.053676559	0.047^a^
Monomethyl glutaric acid	143.84	145.0498	0.225106709	0.194018416	0.036^a^
1-Methylhistamine	61.03	126.1026	0.316643666	0.394875891	0.004^a^
SM (d18:1/14:0)	209.10	675.5447	0.230882341	0.299986299	0.021^a^
Zeranol	31.21	321.1740	0.04441923	0.078249488	0.021^a^
3-(4-Hydroxyphenyl)-1-propanol	34.97	153.0914	0.006442008	0.014148515	0.026^a^
5-Aminopentanamide	82.48	117.1025	0.006249586	0.010608399	0.042^a^
Dihydrouracil	52.30	113.0346	0.030256431	0.05522218	0.021^a^
2-Hydroxypyridine	67.15	96.0448	0.021550707	0.026987291	0.009^a^
3-Amino-2-piperidone	228.28	115.0869	0.012316389	0.025067633	0.041^a^

**b** The information of positive metabolic pathways in two groups			
Pathway	Total	Hits	Hits Cpd
Pantothenate and CoA biosynthesis	27	1	Dihydrouracil
Beta-alanine metabolism	28	1	Dihydrouracil
Glycerophospholipid metabolism	39	1	Choline
Histidine metabolism	44	1	1-Methylhistamine
Lysine degradation	47	1	5-Aminopentanamide
Glycine, serine and threonine metabolism	48	1	Choline
Fructose and mannose metabolism	48	1	Rhamnose
Pyrimidine metabolism	60	1	Dihydrouracil

^*a*^*p* < 0.05, RT: Retention Time, *m*/*z*: Mass-to-Charge Ratio, Mean Grade 4:The mean relative quantification value of the substance in grade 4 group within the group of comparisons, Mean Grade 3:The mean relative quantification value of the substance in grade 3 group within the group of comparisons

Total: Number of metabolites in this pathway, Hits: The number of differential metabolites hitting this pathway, Hits Cpd: Names of differential metabolites hitting this pathway

### Comparison of Metabolic Pathways between the Two Groups

The pantothenate and coenzyme A (CoA) biosynthesis pathway, and the beta-alanine metabolism pathway involving dihydrouracil were significantly different between the two groups. The glycerophospholipid metabolism pathway involving choline, the histidine metabolism pathway involving 1-methylhistamine, the lysine degradation pathway involving 5-aminopentanamide, the glycine, serine and threonine metabolism pathway involving choline, the fructose and mannose metabolism pathway involving rhamnose and the pyrimidine metabolism pathway involving dihydrouracil were also significantly different (Table 2b, and Fig. 2d).

## Discussion

In this study, we used non-targeted metabolomics to analyze the cartilage of knee OA and found that 12 metabolites increased significantly in late-stage knee OA. The related metabolites were choline, 2-hydroxypyridine, 2-propylpiperidine, 3-amino-2-piperidone, 1-methylhistamine, 5-aminopentanamide, 3-(4-hydroxyphenyl)-1-propanol, SM (d18:1/14:0), rhamnose, dihydrouracil, monomethyl glutaric acid, and zeranol. Among them, it has been found that the level of choline is higher in melanoma tumors of a transgenic zebra fish model and dysregulation of glycerophospholipid pathways is related to melanoma metastasis (Henderson et al. 2019). When compared with healthy controls the level of choline was lower in knee OA patients and the glycerophospholipid pathway was differentially activated among healthy, early OA and late OA donor populations (Carlson et al. 2019; Weerasekera et al. 2021). In our study, the level of choline was higher and the glycerophospholipid pathway was differentially activated in grade 4 patients. We may carry out studies to identify how choline regulates the glycerophospholipid pathway in future. Other differently expressed pathways in our study included histidine metabolism involving 1-methylhistamine, lysine degradation pathway involving 5-aminopentanamide and glycine, serine and threonine pathway involving choline. These pathways are rarely studied in OA. We can further explore their correlation with OA in the future, so as to provide a reliable theoretical basis for targeted therapy of OA patients.

Limitations: First, most patients will choose TKA when the imaging findings reach KL 3, while few patients will choose TKA when the imaging findings reach KL 4. So, the number of patients in the two groups is unbalanced. Due to the limitation of inclusion criteria and exclusion criteria, the total samples of the two groups are not large enough. In the future follow-up study, we will continue to increase the sample size to observe confirm our observation in this study. Second, this study focused mainly on knee OA. Whether the findings can be generalized to OA at other joints needs to be established in future study.

## Conclusion

In our study, we combined plain radiography and KL classification to divide 22 knee OA patients into two groups. Cartilages of the femur samples were analyzed using non-targeted metabolomics. We found 12 metabolites and eight metabolic pathways were significantly different between grade 3 and grade 4 patients. This result will provide a reliable basis for targeted metabolomics in future studies of OA. In future studies, we will further explore the specific markers and specific pathways in the articular cartilage of OA patients according to the results of this study, so as to provide accurate evidence for the treatment of OA.

## Supplementary Information

Below is the link to the electronic supplementary material.

Supplementary file1 (PPT 390 KB)

Supplementary file2 (DOC 25 KB)

Supplementary file3 (DOC 20 KB)

## Data Availability

The MS proteomics data have been deposited to the ProteomeXchange Consortium (http://proteomecentral.proteomexchange.org) via the iProX partner repository with the dataset identifier PXD041287.

## Abbreviations

OA: Osteoarthritis
KL: Kellgren–Lawrence
SM: Sphingomyelin
CoA: Coenzyme A
TKA: Total knee arthroplasty
MS: Mass spectrometry
QC: Quality control
LC–MS/MS: Liquid chromatography/tandem MS
UHPLC: Ultra-high-performance liquid chromatography
UPLC: Ultra-performance liquid chromatography
MS2: Secondary mass spectrometry
OPLS-DA: Orthogonal partial least squares-discriminant analysis
PCA: Principal component analysis
VIP: Variable importance in projection
KEGG: Kyoto encyclopedia of genes and genomes
BMI: Body mass index
CT: Computed tomography
MRI: Magnetic resonance imaging
11, 12-DHET: 11,12-Dihydroxyeicosatrienoic acid
14, 15-DHET: 14,15-Dihydroxyeicosatrienoic acid
ALDH1A2: Aldehyde dehydrogenase 1 family member A2
