# Supplementary material for: Identification of Key Factors in Cartilage Tissue During the Progression of Osteoarthritis Using a Non-targeted Metabolomics Strategy
Source: Phenomics. 2024 Mar 10;4(3):227–33. doi: 10.1007/s43657-023-00123-z (PMC11466919; doi:10.1007/s43657-023-00123-z)
Supplement: Supplementary file 1 — Supplementary file1 (PPT 390 KB) [file 43657_2023_123_MOESM1_ESM.ppt]

## Slide 1
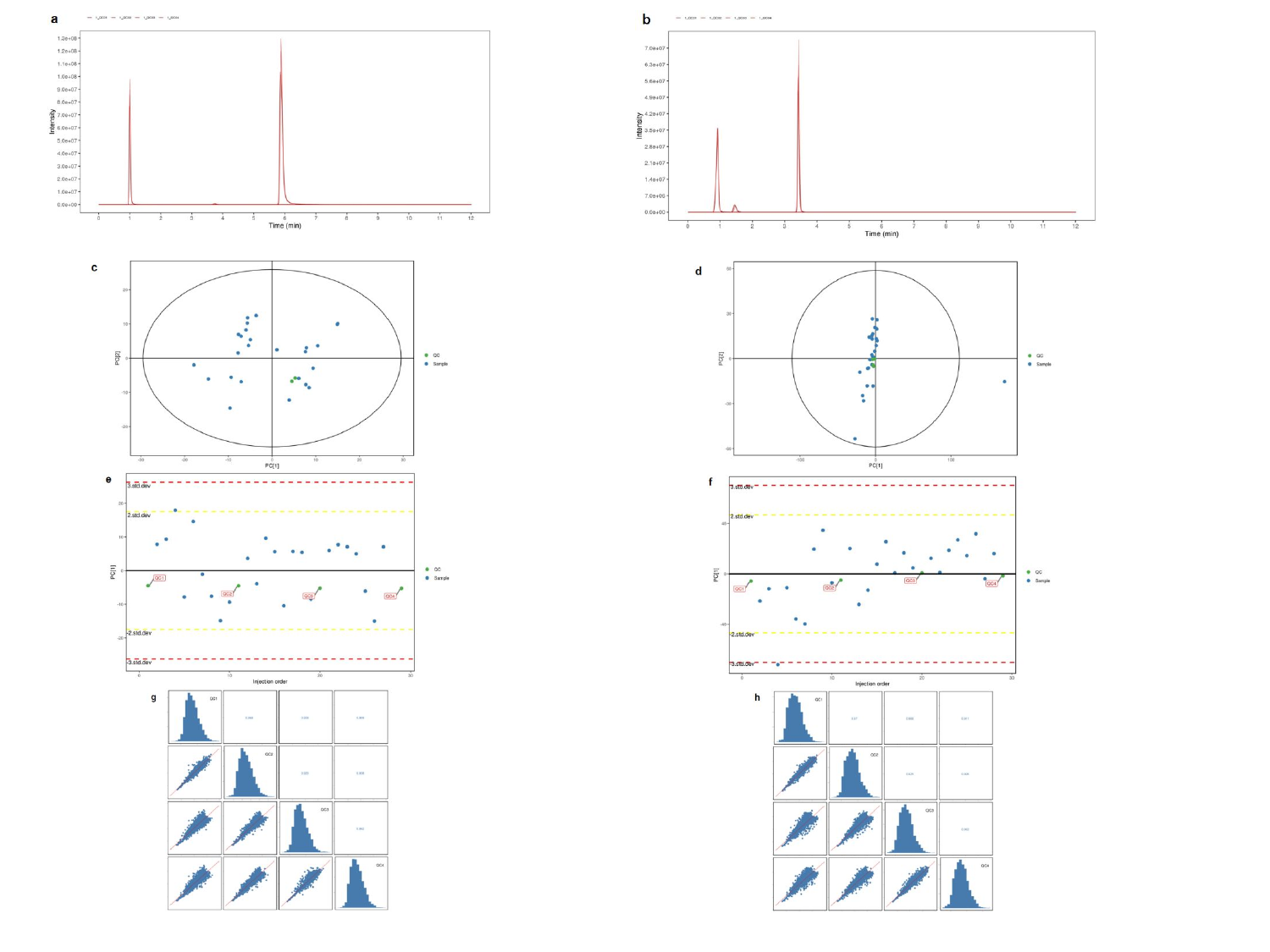

## Slide 2
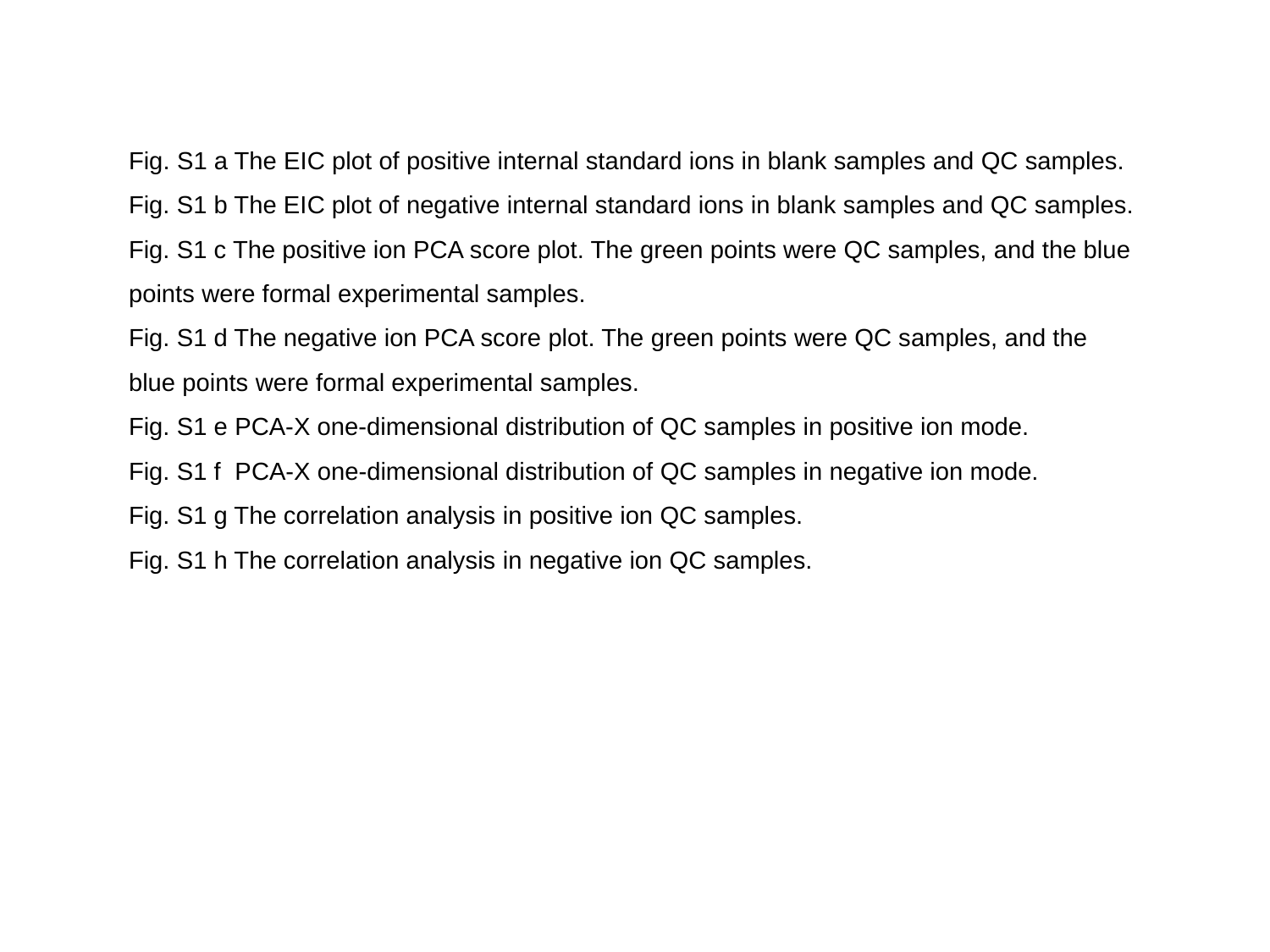

Fig. S1 a The EIC plot of positive internal standard ions in blank samples and QC samples.
Fig. S1 b The EIC plot of negative internal standard ions in blank samples and QC samples.
Fig. S1 c The positive ion PCA score plot. The green points were QC samples, and the blue points were formal experimental samples.
Fig. S1 d The negative ion PCA score plot. The green points were QC samples, and the blue points were formal experimental samples.
Fig. S1 e PCA-X one-dimensional distribution of QC samples in positive ion mode.
Fig. S1 f PCA-X one-dimensional distribution of QC samples in negative ion mode.
Fig. S1 g The correlation analysis in positive ion QC samples.
Fig. S1 h The correlation analysis in negative ion QC samples.
