# Supplementary material for: Identification of Key Factors in Cartilage Tissue During the Progression of Osteoarthritis Using a Non-targeted Metabolomics Strategy
Source: Phenomics. 2024 Mar 10;4(3):227–33. doi: 10.1007/s43657-023-00123-z (PMC11466919; doi:10.1007/s43657-023-00123-z)
Supplement: Supplementary file 2 — Supplementary file2 (DOC 25 KB) [file 43657_2023_123_MOESM2_ESM.doc]

Relevant QC content for QC samples in this study

It could be seen that the retention time and response intensity of the internal standard in the QC samples were stable, indicating that the instrument data acquisition stability was great. It could be seen that no obvious peaks were detected in all internal standards of all blank samples, indicating that substance residues were well controlled, and cross-contamination between samples was controlled within a controllable range (Fig. S1a,b). It could be seen that the QC samples had good aggregation, indicating that the method had good stability (Fig. S1c,d). It could be seen that PCA-X one-dimensional distribution of QC samples were all within ±2 STD, indicating that the data quality of this experiment was very high (Fig. S1e,f). The QC sample correlation was closer to 1, indicating that the stability of the entire method was better and the data quality was higher. We could see that the QC samples were highly correlated, indicating that the data quality of this experiment was very high (Fig. S1g,h). The response difference of the internal standard (median RSD ≤ 15%) was smaller, indicating that the system was more stable and the data quality was higher. We could see that the data quality of this experiment was very high (Table. S1).

Fig. S1 a The EIC plot of positive internal standard ions in blank samples and QC samples.

Fig. S1 b The EIC plot of negative internal standard ions in blank samples and QC samples.

Fig. S1 c The positive ion PCA score plot. The green points were QC samples, and the blue points were formal experimental samples.

Fig. S1 d The negative ion PCA score plot. The green points were QC samples, and the blue points were formal experimental samples.

Fig. S1 e PCA-X one-dimensional distribution of QC samples in positive ion mode.

Fig. S1 f PCA-X one-dimensional distribution of QC samples in negative ion mode.

Fig. S1 g The correlation analysis in positive ion QC samples.

Fig. S1 h The correlation analysis in negative ion QC samples.
