# Supplementary material for: Identification of Key Factors in Cartilage Tissue During the Progression of Osteoarthritis Using a Non-targeted Metabolomics Strategy
Source: Phenomics. 2024 Mar 10;4(3):227–33. doi: 10.1007/s43657-023-00123-z (PMC11466919; doi:10.1007/s43657-023-00123-z)
Supplement: Supplementary file 3 — Supplementary file3 (DOC 20 KB) [file 43657_2023_123_MOESM3_ESM.doc]

**Table S1** Internal standard response stability of QC samples in positive and negative ion mode

| Ion Mode | Sample | RT**(s)** | ***m/z*** | RSD |
| --- | --- | --- | --- | --- |
| positive | IS1 | 352.60 | 85.1327 | 0.0206 |
|  | IS2 | 223.78 | 155.1742 | 0.1278 |
|  | IS3 | 60.15 | 127.0805 | 0.0575 |
| negative | IS1 | 206.58 | 183.0817 | 0.1465 |
|  | IS2 | 56.43 | 158.2012 | 0.0643 |
|  | IS3 | 88.30 | 142.0596 | 0.2245 |

We could see that the data quality of this experiment was very high from this table.
